# Rho kinase cascade activation in circulating leukocytes in patients with diabetes mellitus type 2

**DOI:** 10.1186/s12933-020-01027-2

**Published:** 2020-05-06

**Authors:** Maria Paz Ocaranza, Patricio Valderas, Jackeline Moya, Luigi Gabrielli, Iván Godoy, Samuel Córdova, Paul Mac Nab, Lorena García, Luis Farías, Jorge E. Jalil

**Affiliations:** 1 School of Medicine, Division of Cardiovascular Diseases, Pontificia Universidad Católica de Chile, Diagonal Paraguay 362, Piso 7, 8320000 Santiago, Chile; 2 Center for New Drugs for Hypertension (CENDHY), Pontificia Universidad Católica de Chile, Santiago, Chile; 3 Advanced Center for Chronic Diseases (ACCDiS), Facultad de Medicina, Pontificia Universidad Católica de Chile, Santiago, Chile; 4 Facultad de Medicina, Odontología, Universidad de Antofagasta, Avenida Argentina 2000, 1240000 Antofagasta, Chile; 5 Faculty of Chemical and Pharmaceutical Sciences, Advanced Center for Chronic Diseases (ACCDiS), Universidad de Chile, Santiago, Chile

**Keywords:** Diabetes, Metabolic syndrome, Rho kinase, ROCK, MYPT-1, ERM, Fasudil, JAK, p38 MAPK, Metformin

## Abstract

**Background:**

The intracellular ROCK signaling pathway is an important modulator of blood pressure and of cardiovascular and renal remodeling when Rho-kinase activity is increased. Besides, in preclinical models of diabetes, ROCK activation has also a role in abnormal glucose metabolism as well as in subsequent vascular and myocardial dysfunction. In humans, there are a few data assessing ROCK activation in patients with type 2 diabetes mellitus (T2D) and no studies assessing upstream/downstream components of the ROCK pathway. We assessed here levels of ROCK activation and some of the RhoA/ROCK cascade molecules in peripheral blood mononuclear cells (PBMCs) in T2D patients under current treatment.

**Methods:**

Cross-sectional observational study comparing 28 T2D patients under current antidiabetic treatment with 31 consecutive healthy subjects, matched by age and gender. Circulating levels of malondialdehyde, angiotensin II and inflammatory cytokines IL-6 and IL-8 were determined in all subjects. ROCK activation in PMBCs, upstream and downstream cascade proteins, and levels of the proinflammatory molecules VCAM, ICAM-1 and IL-8 were determined in their PMBCs by Western blot.

**Results:**

Compared to healthy controls, ROCK activation in T2D patients measured by 2 direct ROCK targets in PBMCs was increased by 420 and 570% (p < 0001) and it correlated significantly with serum glucose levels. p38 MAPK phosphorylation (downstream from ROCK) and JAK-2 (upstream from ROCK) were significantly higher in the T2D patients by 580% and 220%, respectively. In T2D patients, significantly increased PBMC levels of the proinflammatory molecules VCAM-1, ICAM-1 and IL-8 were observed compared to control subjects (by 180%, 360% and 260%, respectively). Circulating levels of Ang II and MDA were significantly higher in T2D patients by 29 and 63%, respectively.

**Conclusions:**

T2D patients under treatment with glucose-lowering drugs, antihypertensive treatment as well as with statins have significantly increased ROCK activation in their circulating leukocytes along with higher phosphorylation of downstream cascade proteins despite pharmacologic treatment, along with increased plasma angiotensin II and MDA levels. ROCK inhibition might have an additional role in the prevention and treatment of T2D.

## Background

Upregulated Rho kinase (ROCK) activity is involved in the pathogenesis of all aspects of the metabolic syndrome [1, 2]. The ROCK pathway or cascade is an intracellular mechanism that triggers cardiovascular remodeling with important roles in blood pressure regulation, vascular smooth muscle contraction, and cardiovascular and renal remodeling. The small RhoA protein is activated by agonists of receptors coupled to the cell membrane G protein, such as angiotensin II, noradrenaline, growth factors or by cytokines [3–5]. Once RhoA is activated, it translocates to the cell membrane where it activates ROCK. Activated ROCK plays a significant role in vascular smooth muscle cell contraction and regulates several cellular functions involved in cardiovascular remodeling (such as actin cytoskeleton organization, adhesion and motility, proliferation, differentiation, apoptosis and cell survival). ROCK also mediates upregulation of several proinflammatory, thrombogenic and fibrogenic molecules and downregulation of endothelial nitric oxide synthase [3, 4]. In this way, RhoA and ROCK activation have important effects on several cardiovascular diseases [3–6]. In humans with metabolic syndrome, ROCK activation determined in circulating leukocytes has been observed in 2 studies [2, 7].

Besides, in experimental models of metabolic syndrome and type 2 diabetes mellitus (T2D), ROCK activation has a role both in abnormal glucose metabolism pathophysiology as well as in subsequent vascular and myocardial dysfunction. Pharmacological ROCK inhibition increases the number of L cells in the gut, plasma levels of Glucagon-like peptide 1 (GLP1) and insulin and reduces blood glucose levels in mice [8]. Additionally, in mice with insulin resistance, ROCK inhibition increases secretion of GLP1 and glucose tolerance compared with saline administration [8]. Increased ROCK activity has been found in the skeletal muscle and vasculature of insulin-resistant Zucker obese rats and ROCK inhibition prevents the development of various aspects of metabolic syndrome including hypertension, insulin resistance, dyslipidemia, and obesity [9]. In streptozotocin induced diabetic mice, enhanced ROCK activity and decreased nitric oxide bioavailability contribute to altered reactivity to acetylcholine and Ang II in basilar arteries [10]. Furthermore, long-term administration of the ROCK inhibitor fasudil to diabetic rats, significantly ameliorated diabetes-induced cardiac contractile dysfunction at cellular and whole organ levels, specifically fasudil improved calcium removal in diabetic cardiomyocytes, normalized the G/F-actin ratio and promoted F-actin organization [11].

However, in humans there are a few data assessing ROCK activation in patients with T2D. Besides, there are no human studies assessing other components of the ROCK cascade (upstream or downstream) in T2D patients. In Chinese T2D patients under treatment, Liu et al. recently found that ROCK activity was significantly increased in circulating leukocytes [12] compared to control subjects. In a pilot study in patients with T2D and preserved left ventricular ejection fraction, short term fasudil administration improved echocardiographic parameters of left ventricle (LV) diastolic function, but ROCK activation in those patients was not reported [13]. Thus it is relevant to assess in another T2D population, ROCK activation levels simultaneously with proteins of the RhoA/ROCK cascade and with LV function.

The aim of this study was to assess levels of ROCK activation and some of the RhoA/ROCK cascade molecules in peripheral blood mononuclear cells (PBMCs) in type 2 diabetic patients under current treatment and its relationship with LV function and levels of Angiotensin II.

## Methods

### Study population

This cross-sectional observational study included consecutive T2D patients (n = 28), under current treatment with glucose-lowering drugs not changed during the previous 12 weeks, nonobese (body mass index < 27 kg/m^2^) and serum creatinine < 1.4 mg/dl and. All of them were also hypertensive patients controlled by antihypertensive treatment. Their clinical characteristics and the current medical treatment are depicted in Tables 1 and 2, respectively. As controls, 31 consecutive healthy subjects (by medical history, symptoms, electrocardiogram, and echocardiogram), matched by age and gender, not taking antidiabetic or antihypertensive drugs, were included. Exclusion criteria were neoplastic disease in the last 4 years, active infection in the last 8 weeks, and chronic lung, liver or kidney disease.

**Table 1:** Demographics, blood pressure and heart rate

	Control subjects (n = 31)	T2D patients (n = 28)	*p*
Age (years)	57.8 ± 5.5	60.7 ± 5.4	0.080
Men (%)	34	58	0.150
Weight (Kg)	68.4 ± 8.6	70.1 ± 10.7	0.500
Body mass index (BMI, Kg/m^2^)	25.3 ± 2.3	26.4 ± 2.1	0.070
Systolic blood pressure (mm Hg)	114.1 ± 12.0	123.8 ± 16.0	0.010
Diastolic blood pressure (mm Hg)	75.4 ± 7.7	81.0 ± 9.0	0.010
Heart rate (bpm)	65.1 ± 8.9	73.0 ± 9.0	0.001

Values are shown as mean ± SD

**Table 2:** Current pharmacological treatment in the T2D patients (n = 28)

Pharmacological treatment	
Metformin (%)	89
Only metformin (%)	14
Other oral hypoglicemic drug (%)	43
Metformin + Other oral hypoglicemic drug (%)	38
Insulin (%)	57
Only insulin (%)	4
Insulin + Metformin (%)	50
Insulin + any oral hypoglicemic drug (%)	54
ACE inhibitors (%)	21
Angiotensin receptor blockers (%)	75
Betablockers (%)	36
Thiazides (%)	25
Statins (%)	71
Aspirin (%)	43

The study was approved by the Human Research Ethics Committee at the Pontificia Universidad Catolica de Chile, School of Medicine and adhered to the Declaration of Helsinki principles. Written informed consent was obtained from all participants prior to any procedure.

### Biochemical parameters

Fasting concentrations of total cholesterol, triglycerides (TG), low-density lipoprotein cholesterol (LDL-C), high density lipoprotein cholesterol (HDL-C), plasma glucose, and glycated hemoglobin A1c (HbA1c) were determined at the Central Clinical Laboratory, Red Christus UC.

### Echocardiographic measurements

Echocardiograms were obtained with a 2.5-MHz transducer at the time of blood sampling with a Phillips IE-33 instrument (Andover, MA) to evaluate LV function, geometry, and mass. All measurements were performed blindly as previously described [14] and according to the recommendations of the American Society of Echocardiography [15]. The following variables in the parasternal short axis were measured: left atria diameter, interventricular septal thickness and posterior wall thickness, end diastolic dimension and end-systolic dimension. Ejection fraction was determined by the Simpson method. Systolic pulmonary artery pressure was assessed by continuous wave Doppler of the tricuspid regurgitation trace, to measure the difference in pressures between the right ventricle and right atrium and using the simplified Bernoulli equation to calculate this pressure difference using peak tricuspid regurgitation velocity [16].

### Plasma levels of malondialdehyde

As one parameter associated to oxidative stress, we measured circulating malondialdehyde levels as previously described by determining the content of thiobarbituric acid-reactive substances [17].

### Protein extraction from peripheral blood mononuclear cells (PBMCs)

For isolating peripheral blood mononuclear cells (PBMCs), 5 mL of whole venous blood containing ethylenediaminetetraacetic acid (EDTA) were poured over 5 mL of density gradient cell separation medium (Ficoll and sodium diatrizoate, Histopaque-1077, Sigma Chemical Co., St Louis, MO) and centrifuged [14, 18]. White cells were separated, resuspended and washed in phosphate buffered saline (PBS). Upon isolation (4–80 × 10^6^ viable cells, 95% viability), cells were resuspended in lysis buffer containing 150 mM NaCl, 1% NP40, 0.5% deoxycholate, 0.1% sodium dodecyl sulfate (SDS) and 50 mM Tris. Lysis buffer was supplemented with a protease inhibitor cocktail (1 μg/ml aprotinin, 1 μg/ml leupeptin and 1 mM phenylmethylsulphonyl fluoride) [14, 18]. Protein content was determined by the Lowry assay. ROCK activity and ROCK cascade proteins were determined by Western blot (details as follows).

### Western blot analysis to determine ROCK activity and ROCK cascade proteins in PBMCs

ROCK activity was measured as the relationship of phosphorylated to total myosin phosphatase target subunit 1 (MYPT-1) and also by the relationship of phosphorylated to total ezrin, radixin, moesin (ERM), two direct ROCK substrates.

Soluble protein fractions were heated 5 min at 95 °C with SDS sample buffer (375 mM Tris–HCl pH 6.8, 6% SDS, 48% glycerol, 9% 2-mercaptoethanol and 0.03% bromophenol blue). Equal amounts of protein were loaded and separated on a 5% stacking and 8, 18% resolving SDS-PAGE gel (80 V), and transferred into a nitrocellulose membrane at 400 A during 2 h on ice. Blocking was performed with 5% BSA at room temperature. Blots were incubated overnight at 4 °C with the primary antibody. The relative amount of protein was estimated by chemiluminescence (ECL plus kit, Perkin Elmer which contains the substrate for horseradish peroxidase, HRP). Digital images were obtained with a Syngene G-Box automated system and analyzed by densitometry using the software UN-SCAN-IT™ (Silk Scientific Corporation).

The blots were incubated overnight with the following primary antibodies: anti myosin phosphatase target subunit 1 (anti-MYPT-1 antibody, rabbit polyclonal, 1/500 cell signaling, Cat 2634); anti-p-MYPT-1 antibody (phospho-MYPT1-Thr 853 rabbit polyclonal, 1/500, cyclex, Cat CY-P1025) [14, 16]; anti ezrin, radixin, moesin (anti-ERM antibody, total-ERM, rabbit polyclonal, 1/700, Cell Signaling, Cat 3142); anti-p-ERM antibody (phospho-ERM, ezrin Thr567, radixin Thr564, moesin Thr558, rabbit polyclonal, 1/700, Cell Signaling, Cat 3141); anti p-38 mitogen-activated protein kinase (anti-p38 MAPK antibody, p-38, rabbit polyclonal, 1/1000, Cell Signaling, Cat 9212); anti-phospho-p38 MAPK antibody (Thr180/Tyr182) (phospho-p-38, rabbit monoclonal, 1/1000, Cell Signaling, Cat 4511); anti-ROCK-1 antibody (mouse monoclonal, 1/500, BD Bioscience, Cat 611136); anti-ROCK-2 antibody (mouse monoclonal, 1/2000, BD Bioscience, Cat 610623); anti-p65 nuclear factor κB antibody (NFκB, rabbit polyclonal, 1/1000, Cell Signaling, Cat 8242); anti-vascular cell adhesion molecule 1 antibody (VCAM-1, goat polyclonal, 1/1000, Santa Cruz, Cat sc1504); anti-intracellular adhesion molecule 1 antibody (ICAM-1, mouse monoclonal, 1/1000, Santa Cruz, Cat sc8439); anti-interleukin 6 antibody (IL-6, rabbit polyclonal, 1/500, Abcam, Cat ab6672); anti-interleukin 8 antibody (IL-8, rabbit polyclonal, 1/500, Abcam, Cat ab7747); anti-myosin light chain 2 antibody (MLC-2, rabbit polyclonal, 1/1000, Cell Signaling, Cat cs3672) and anti-phospho-MLC-2 antibody (Thr18/Ser19) (p-MLC-2, rabbit polyclonal, 1/500, Cell Signaling, Cat cs3674); anti Janus kinase (anti JAK antibody, JAK, rabbit polyclonal, 1:1000 Cell signaling Cat cs3230); anti-phospho JAK2 antibody (p-JAK, 1:500 Rabbit polyclonal Cell signaling Cat sc3776); anti JNK antibody (JNK, rabbit polyclonal, 1:1000 cell signaling cs9252) and anti phospho c-Jun N-terminal kinase (anti-phospho JNK antibody, p-JNK, 1:500 rabbit polyclonal Cell signaling cat cs9251). The blots were then washed and incubated with a secondary antibody horseradish peroxidase conjugated goat anti-rabbit IgG (1:7500, Thermo Scientific, Cat) or a goat anti-mouse IgG (1:10.000, Santa Cruz, Cat sc2005) for 2 h. As a protein loading control, β-actin (β-actin, mouse monoclonal, 1/10,000, Sigma, Cat A2228) was used.

### Circulating levels of angiotensin II and inflammatory cytokines IL-6 and IL-8

Venous blood samples were taken and immediately cooled, centrifuged at 4 °C and then stored at − 80 °C until analysis. Angiotensin II (Ang II) levels were quantified using commercial ELISA kits (Sigma, US; Enzo Life Sciences) according to the manufacturer instructions. The Ang II ELISA detection limits were 4.6 pg/ml with intra-assay coefficient of variation (CV) between 4.7 and 7.3% and inter-assay CV between 6.0 and 15.9%. Serum levels of IL-6 and IL-8 were also determined using the Abcam ELISA kit according to the manufacturer’s instructions.

### Statistical analysis

Data are presented as mean ± SD. Differences between mean values were compared using t-test. Correlation analyses were performed by using Pearson correlation. p Values ≤ 0.05 were considered statistically significant.

## Results

### Clinical characteristics and laboratory tests

Twenty-eight consecutive patients with T2D with a mean diabetic history of 133 months and a mean HbA1C 7.9 ± 1.1% were included in the study. In the control group 31 consecutive healthy subjects without heart disease assessed by medical history, symptoms, electrocardiogram and echocardiogram were included. Age, weight, BMI and gender distribution were similar in both groups (Table 1). Blood pressure and heart rate were slightly higher in the T2D patients (Table 1). 96% of the T2D patients were receiving oral glucose-lowering drugs (most of them metformin) and 54% were treated with an oral glucose-lowering drug plus insulin (Table 2).

Compared to the control group, serum creatinine and potassium, triglycerides and SGOT levels were similar in T2D patients, but most of blood chemistry tests as well as levels of malondialdehyde were significantly different (p < 0.05) (Table 3).

**Table 3:** Blood chemistry and plasma oxidative stress levels

	Control subjects (n = 31)	T2D patients (n = 28)	*p*
Hematocrit (%)	42.9 ± 3.4	40.8 ± 3.4	0.020
Hemoglobin (mmol/L)	14.5 ± 1.4	13.8 ± 1.3	0.040
WBC/Ml	6003.2 ± 1461.4	7465.9 ± 1733.8	0.020
Plasma creatinine (mEq/L)	0.8 ± 0.2	0.8 ± 0.2	0.690
ESR	6.5 ± 5.9	13.0 ± 15.9	0.030
BUN (mg/L)	13.2 ± 4.8	16.7 ± 4.1	0.030
Potassium, serum (mEq/L)	4.3 ± 0.7	4.3 ± 0.4	0.680
Glucose, serum (mg/dL)	89.2 ± 6.71	155.0 ± 59.2	0.001
Uric acid (μmol/L)	5.1 ± 1.2	4.4 ± 1.4	0.040
BUN/plasma creatinine	17.7 ± 3.4	22.2 ± 5.7	0.010
Serum Cholesterol (mg/dL)	204.5 ± 22.5	160.1 ± 42.3	0.001
LDL Cholesterol (mg/dL)	124.4 ± 23.9	87.2 ± 34.7	0.001
HDL Cholesterol (mg/dL)	55.7 ± 14.3	44.1 ± 9.4	0.001
Triglicerydes (mg/dL)	127.0 ± 63.3	144.1 ± 72.9	0.340
SGOT (U/L)	21.2 ± 6.3	19.7 ± 6.1	0.350
MAU (ug/mL)	2.2 ± 3.0	23.6 ± 40.77	0.010
MAU/urine creatinine ratio (mg/g creat)	4.9 ± 1.8	28.3 ± 50.6	0.030
MDA (uM)	1.1 ± 0.4	1.8 ± 1.1	0.010

Values shown as mean ± SD

*NS* nonsignificant, *WBC* white blood cells, *BUN* blood urea nitrogen, *MDA* malondialdehyde, *HDL-C* density lipoprotein cholesterol, *MAU* microalbuminuria

### Echocardiographic LV dimensions and function

Compared with the control group, posterior wall thickness, septum thickness, and A wave velocity were significantly larger in the T2D patients by 8%, 12%, and 15%, respectively (Table 4). LV systolic function was normal in the T2D patients, whereas LV diastolic function assessed by the the E/A ratio was reduced (p < 0.02; Table 4).

**Table 4:** Cardiac dimensions, LV systolic function and pulmonary artery systolic pressure by echocardiography

	Control subjects (n = 31)	T2D patients (n = 28)	*p*
Posterior wall thickness (mm)	8.7 ± 1.0	9.4 ± 1.3	0.020
Septum thickness (mm)	9.0 ± 1.0	10.1 ± 1.5	0.001
LV end diastolic diameter (mm)	48.8 ± 4.5	45.3 ± 4.7	0.001
LV end systolic diameter (mm)	30.1 ± 4.7	28.0 ± 4.5	0.090
LV end diastolic diameter (mm)/BMI	1.9 ± 0.4	1.7 ± 0.2	0.120
LV end systolic diameter (mm)/BMI	1.1 ± 0.3	1.1 ± 0.2	0.220
LV end diastolic diameter (mm)/height (cms)	0.3 ± 0.1	0.3 ± 0.0	0.930
LV end sytolic diameter (mm)/height (cms)	0.2 ± 0.0	0.2 ± 0.0	0.810
Left atrial diameter (mm)	37.2 ± 5.0	38.8 ± 4.1	0.150
LV mass (g)	189.0 ± 44.3	192.5 ± 48.9	0.780
LV mass index (g/m^2^)	103.5 ± 20.4	111.2 ± 24.6	0.210
LV shortening fraction (%)	38.1 ± 7.1	37.8 ± 7.2	0.810
LV ejection fraction (%)	67.4 ± 5.0	64.0 ± 7.3	0.070
Pulmonary artery systolic pressure (mm Hg)	25.4 ± 3.8	25.0 ± 4.2	0.770
E wave velocity, cm/s	70.1 ± 14.0	71.8 ± 10.8	0.600
A wave velocity, cm/s	69.1 ± 18.0	79.8 ± 15.4	0.020
E/A	1.0 ± 0.2	0.9 ± 0.2	0.010

Values shown as mean ± SD

*LV* left ventricle, *E/A* transmitral filling waves ratio

### Rho-kinase activation in PBMC cells

**Fig. 1:**
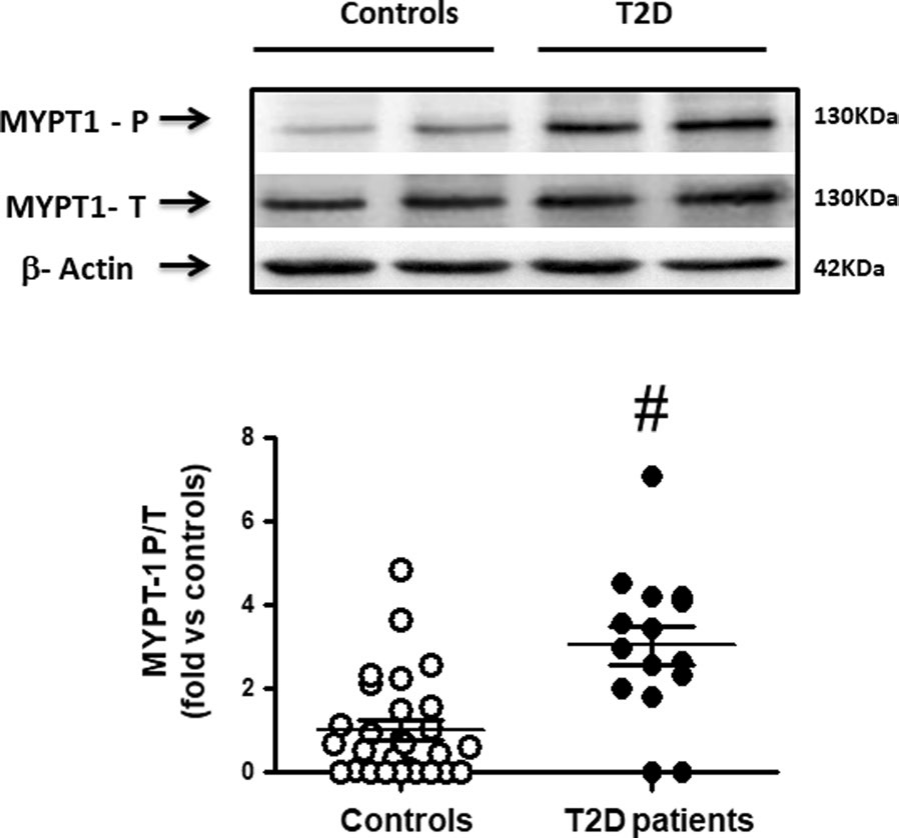
Increased ROCK activation in PBMCs assessed by MYPT-1 phosphorylation levels in T2D patients (Western blot). Upper panel. Representative western blots images for phosphorylated (MYPT1 P) and total MYPT1 (MYPT1 T) in PBMCs from 2 control subjects and 2 T2D patients. Lower Figure. Dot graph of phosphorylated/total MYPT1 ratios in PBMCs in Control subjects (n = 28, white circles) and in T2D patients (n = 15, black circles), data shown as mean ± SEM. Symbol: ^#^p < 0,001 versus Controls

**Fig. 2:**
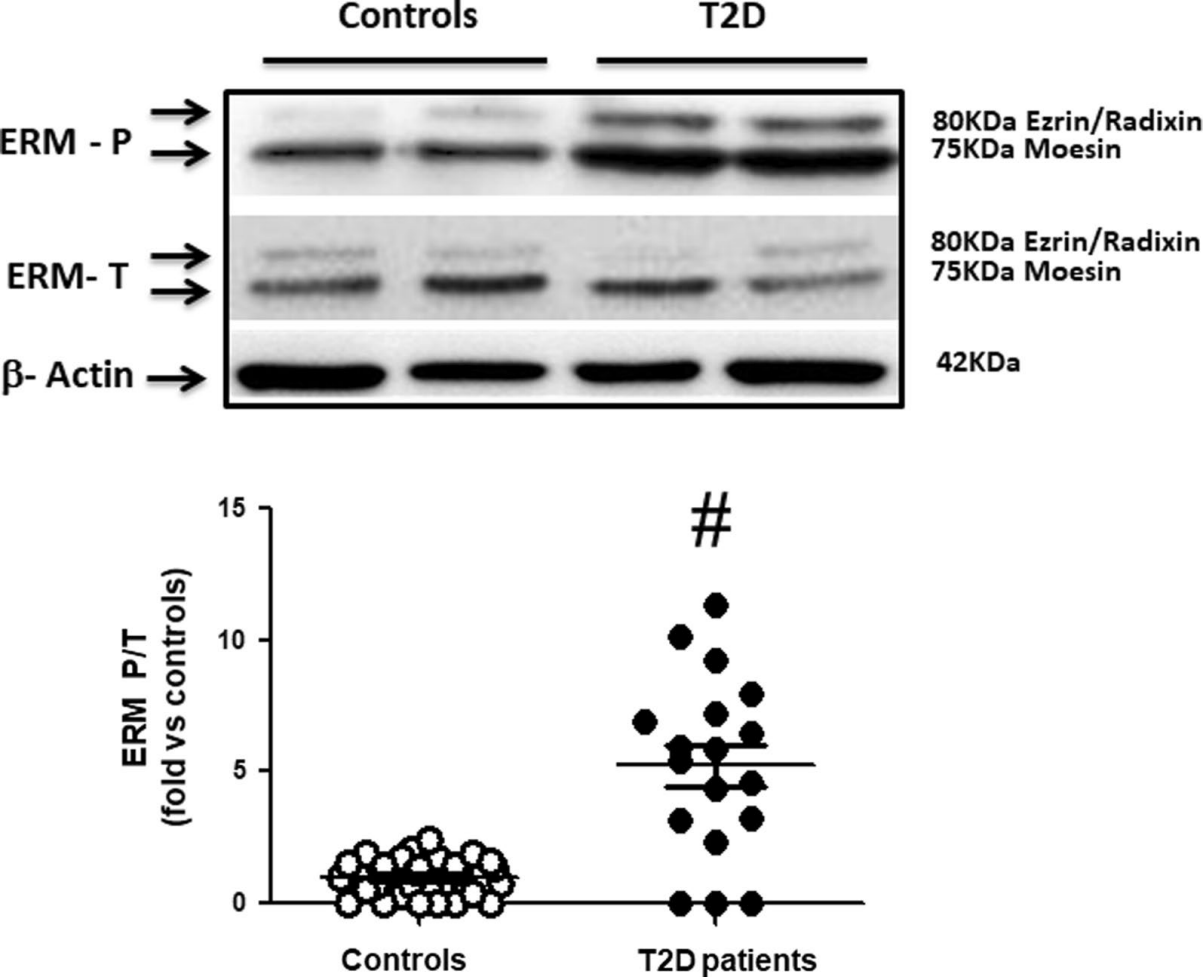
Increased ROCK activation in PBMCs assessed by ERM phosphorylation in T2D patients (Western blot). Upper panel. Representative western blots images for phosphorylated (ERM P) and total ERM (ERM T) in PBMCs from 2 control subjects and 2 T2D patients. Lower Figure. Dot graph of phosphorylated/total ERM ratios in PBMCs in Control subjects (n = 31, white circles) and in T2D patients (n = 18, black circles), data shown as mean ± SEM. Symbol: ^#^p < 0,001 versus Controls

**Fig. 3:**
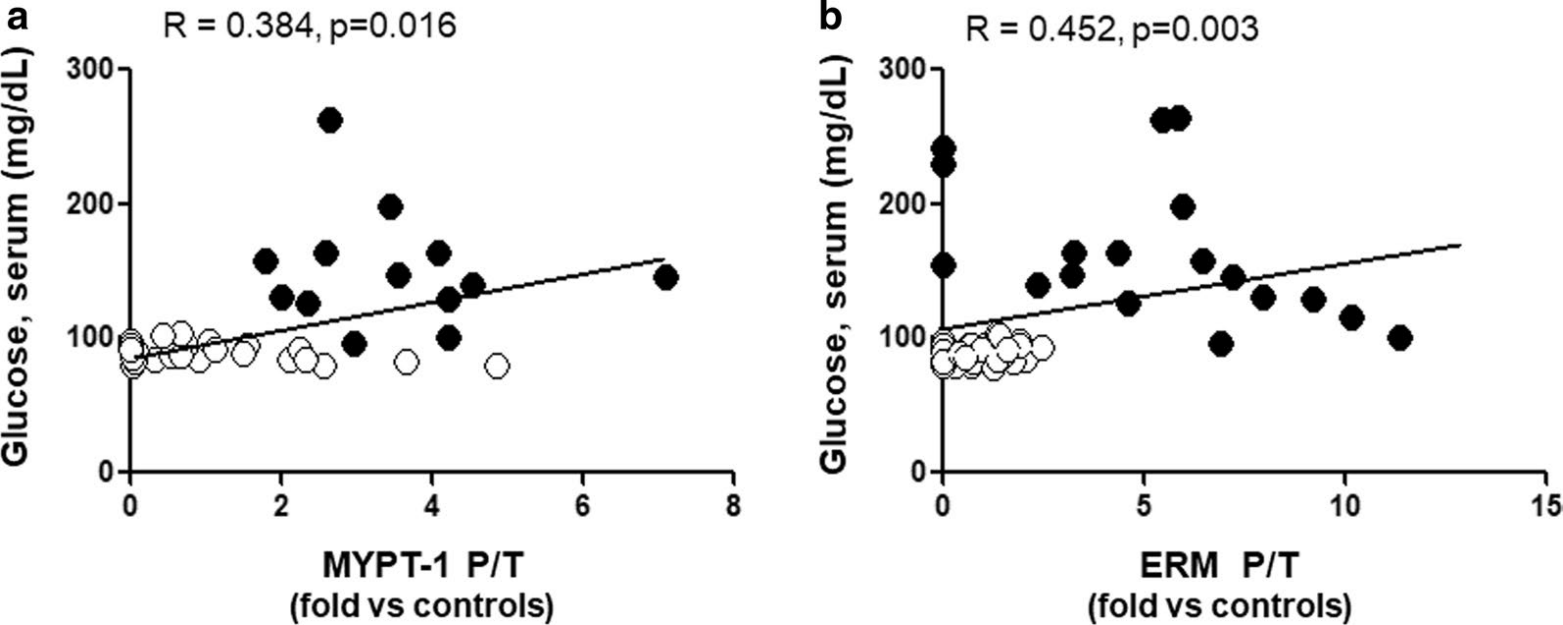
Linear relationship (Pearson coefficient) between serum glucose levels and ROCK activation in PBMCs assessed both by MYPT-1 (**a**), n = 39, and ERM phosphorylation levels (**b**), n = 41. White circles = Control subjects, black circles = T2D patients

In the T2D patients, the mean ratio between phosphorylated to total MYPT1 and ERM (MYPT1-P/T and ERM-P/T) in circulating leukocytes, was significantly increased by threefolds (Fig. 1, p < 0.001) and fivefolds (Fig. 2, p < 0.001), respectively, compared to control subjects. MYPT1-P/T and ERM-P/T ratios were directly correlated with serum glucose levels (r = 0.38, p < 0.02, Fig. 3a) and (r = 0.45, p < 0.004), respectively (Fig. 3b).

**Fig. 4:**
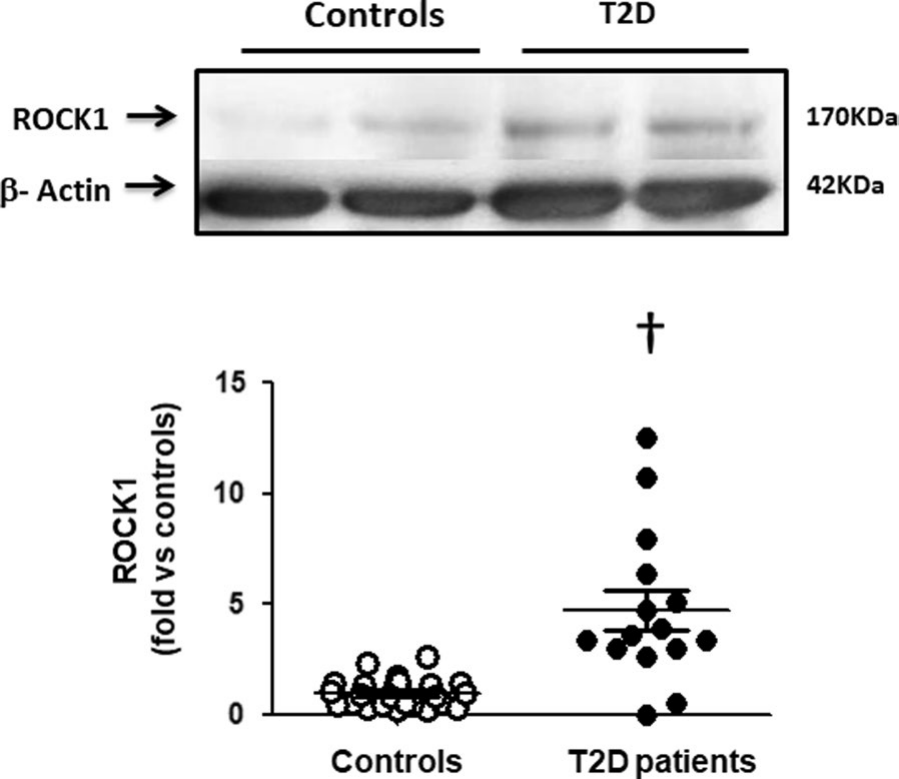
Increased ROCK1 isoform levels in PBMCs in T2D patients (Western blot). Upper panel. Representative western blots images of ROCK1 isoform in PBMCs from 2 control subjects and 2 T2D patients. Lower Figure. Dot graph comparing ROCK1 isoform levels in PBMCs in Control subjects (n = 25, white circles) and in T2D patients (n = 15, black circles), data shown as mean ± SEM. Symbol: ^†^p < 0,002 versus controls

Besides, ROCK1 levels in PBMCs were significantly higher in the T2D patients compared to healthy subjects (p < 0.002, Fig. 4), whereas similar levels of the ROCK 2 isoform were observed in both groups (Table 5).

**Table 5:** Components of the ROCK pathway in PBMCs (Western blot)

	Control subjects	T2D patients	*p*
p38-MAPK P/T	1.00 ± 0.90	5.81 ± 3.50	0.001
p65-NFkB (UOD)	1.00 ± 0.45	1.34 ± 1.18	0.240
JAK2 P/T	1.00 ± 0.68	2.28 ± 2.10	0.052
ROCK2 (UOD)	1.00 ± 0.57	0.68 ± 0.65	0.080
JNK P/T	1.00 ± 0.63	0.90 ± 1.16	0.960
IL-6 (UOD)	1.00 ± 1.00	1.03 ± 0.75	0.920
MLC-2 P/T	1.00 ± 0.63	0.98 ± 1.16	0.960

Values are shown as mean ± SD (fold vs control subjects) n = 22-27 Controls and 13 -18 T2D patients

*p38-MAPK* p38 mitogen-activated protein kinase, *p65-NFkB* Nuclear factor NF-kappa-B p65 subunit, *JAK2* Janus kinase 2, *ROCK2* Rho kinase isoform 2, *JNK* Jun amino-terminal kinase, *IL-6* interleukin 6, *MLC-2* myosin light chain 2, *P/T* phosphorylated/total, *UOD* units of optical density

### Components of the ROCK cascade in circulating leukocytes

In the T2D patients JAK2 phosphorylation levels (upstream from ROCK) were increased by 2.2-fold and p38 MAPK phosphorylation levels (downstream from ROCK) were significantly increased by and 5.8-fold (Table 5) in their PBMCs.

**Fig. 5:**
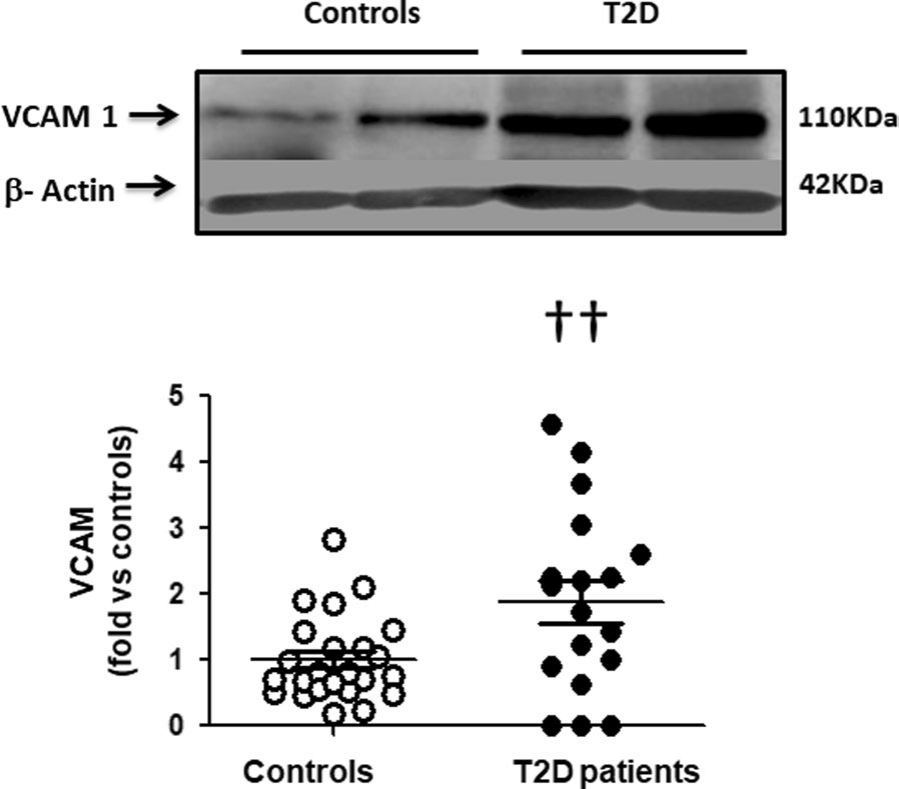
Higher VCAM-1 levels in PBMCs in T2D patients compared to control subjects (Western blot). Upper panel. Representative western blots images of VCAM in PBMCs from 2 control subjects and 2 T2D patients. Lower Figure. Dot graph comparing VCAM levels in PBMCs in Control subjects (n = 24, white circles) and in T2D patients (n = 18, black circles), data shown as mean ± SEM. Symbol: ^††^p < 0,05 versus controls

**Fig. 6:**
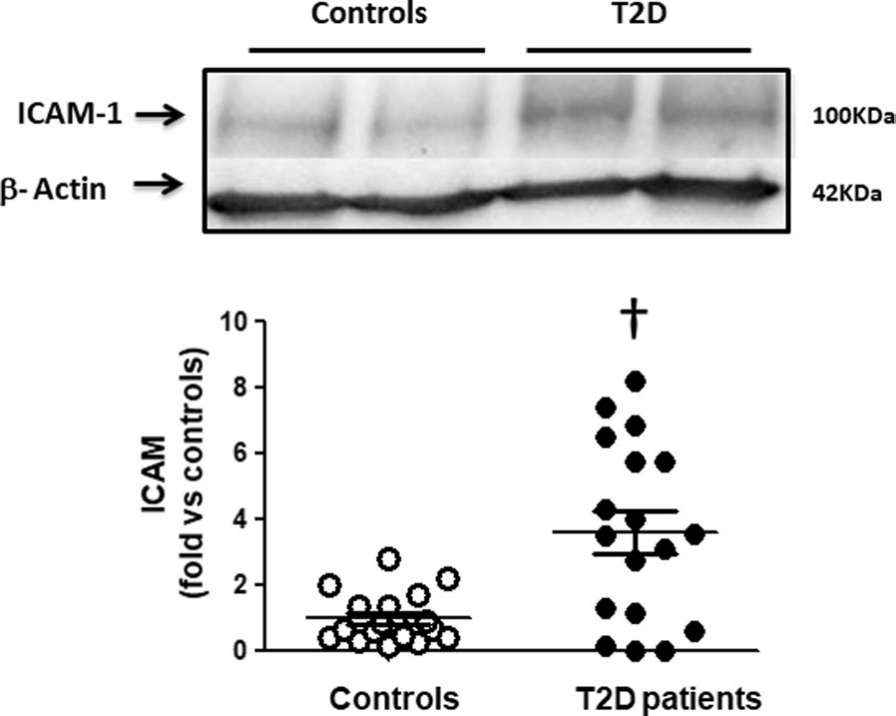
Increased ICAM-1 levels in PBMCs in T2D patients compared to control subjects (Western blot). Upper panel. Representative western blots images of ICAM in PBMCs from 2 control subjects and 2 T2D patients. Lower Figure. Dot graph comparing ICAM levels in PBMCs in Control subjects (n = 18, white circles) and in T2D patients (n = 18, black circles), data shown as mean ± SEM. Symbol: ^†^p < 0,002 versus Controls

**Fig. 7:**
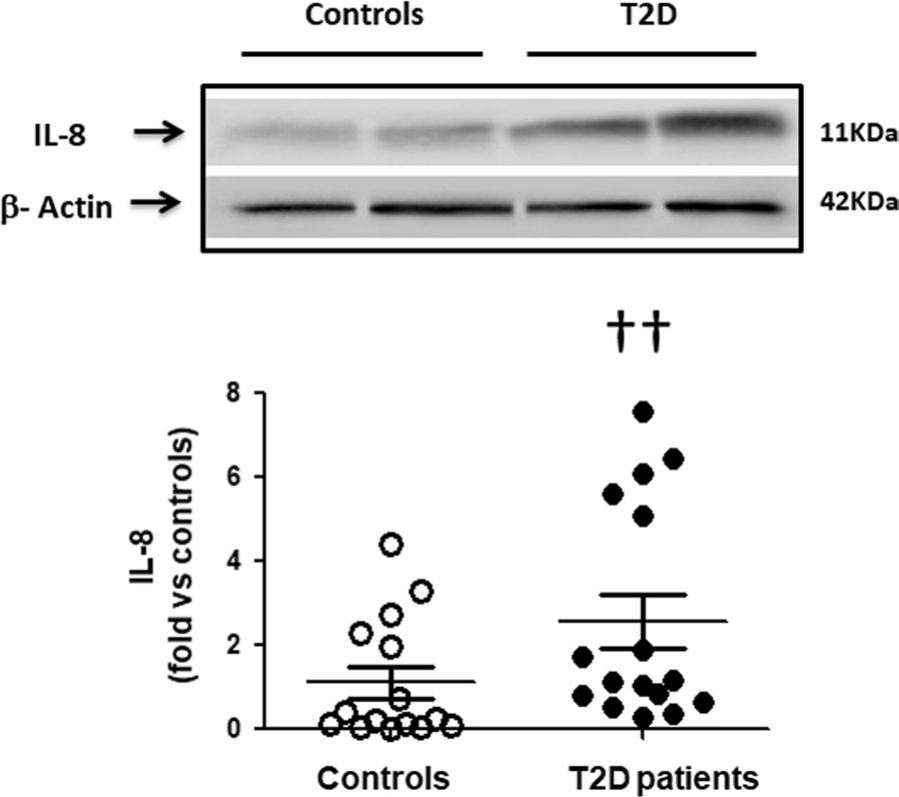
Increased IL-8 levels in PBMCs in T2D patients compared to control subjects (Western blot). Upper panel. Representative western blots images of IL-8 in PBMCs from 2 control subjects and 2 T2D patients. Lower Figure. Dot graph comparing IL-8 levels in PBMCs in Control subjects (n = 20, white circles) and in T2D patients (n = 16, black circles), data shown as mean ± SEM. Symbol: ^††^p < 0.05 versus Controls

Furthermore, a significant increase in the levels of VCAM-1 (by 1.8-fold, p < 0.05, Fig. 5), ICAM-1 (by 3.6-fold, p < 0.002, Fig. 6) and IL-8 (by 2.6-fold, p < 0.05, Fig. 7) were observed in the diabetic patients compared to control subjects.

### Circulating levels of Angiotensin II, IL-8 and IL-6

Even though the T2D patients were receiving pharmacological treatment and all of them were clinically stable, circulating levels of Ang II were significantly higher in patients compared to controls by 29% (Table 6). Circulating levels of ILs 6 and 8 were similar in both groups (Table 6).

**Table 6:** Circulating levels of interleukins 6 and 8 and angiotensin II

	Control subjects (n = 31)	T2D patients (n = 28)	*p*
Interleukin-6 (pg/mL)	0.95 ± 0.31	1.77 ± 0.52	0.180
Interleukin-8 (pg/mL)	4.89 ± 0.66	5.23 ± 1.08	0.780
Angiotensin II (pg/mL)	7.78 ± 2.80	10.08 ± 1.38	0.040

Values shown as mean ± SD; n = 10–30 Controls and 9–28 T2D patients

## Discussion

The main findings in this study were that T2D patients under current glucose-lowering drugs compared with healthy control subjects have significant ROCK activation in their PBMCs as determined by increased phosphorylation of two ROCK direct targets (MYPT1 and ERM). Besides, increased activity of the ROCK cascade proteins JAK-2 (upstream) and p38-MAPK (downstream) were found. ROCK activation levels were here correlated with current blood glucose levels. In these patients, increased levels of the proinflammatory molecules VCAM, ICAM-1 and IL-8 and of the isoform ROCK1 were also found in their PMBCs despite pharmacologic treatment along with increased plasma angiotensin II and MDA levels (by 30% and 64%, respectively).

There is only one clinical study in T2D patients under treatment (26% of them were also hypertensives) observing significantly higher levels of ROCK activation in circulating leukocytes by 11% compared to controls [12] and our study is consistent with those observations, with larger significant differences in ROCK activation in our current study assessed by 2 different ROCK substrates phosphorylation. In both studies a significant relationship of ROCK activation in PBMCs with glucose levels has been observed.

Higher ROCK activation levels in the present study, were observed despite that 96% of the patients were receiving ACE inhibitors/ARBs and their blood pressure was normal but higher than in control subjects. In hypertensive patients, olmesartan [19, 20] reduces ROCK activation levels in PBMCs, as well as amlodipine [21] and eplerenone [22]. Consequently, it is possible to hypothesize that chronically elevated blood glucose levels may explain both higher ROCK activation levels and increased plasma angiotensin II levels in treated T2D patients (increased angiotensin levels II are associated with ROCK activation in PMBCs in normotensive rats with genetically induced high ACE levels and angiotensin II and in patients with heart failure) [23, 24].

The specific biological implication of ROCK activation in PMBCs in diabetic patients, as in patients with cardiovascular diseases, is not totally known. Our understanding of the pathophysiology of ROCK activation, downstream ROCK pathway and the related molecular mechanisms involved in its pathogenesis and how does activated ROCK in leukocytes affect each target organ is even less known [25]. In this regard, two preclinical studies assessing simultaneously ROCK activation in PMBCs, myocardium and in the aortic wall indicate a direct relationship between ROCK activation in PMBCs and in cardiovascular tissue, supporting the concept that ROCK activation in these circulating cells does reflect cardiovascular ROCK activation [23, 26].

In PBMCs, we observed here increased ROCK1 isoform levels in T2D patients and unchanged levels of the ROCK2 isoform. Patients with hypertension and patients with heart failure and reduced ejection fraction have similar ROCK isoform levels in their PBMCs [14, 18, 24]. There are no previous ROCK isoform measurements in humans with T2D but patients with metabolic syndrome have similar ROCK isoform levels compared to control subjects [7]. In non diabetic mice, double ROCK knockout promotes cardiac autophagy and fibrosis, whereas in ROCK2 knockout mice, autophagy is inhibited and increased cardiac fibrosis is observed [27]. At the same time loss of ROCK1 does not have an effect on autophagy [27]. In diabetic hearts from streptozotocin treated rats, ROCK activation is associated with over-expression of the ROCK2 isoform [28]. Cardiomyocytes from diabetic mice develop arrhythmic calcium transients in response to increased [Ca^2+^] which is attenuated in cardiomyocytes from diabetic ROCK2 mice [29] suggesting that ROCK2 contributes to diabetes-induced impaired cardiac calcium homeostasis. It is possible that ROCK2 may be a beneficially therapeutic target for heart protection in T2D [30], which needs further preclinical and clinical research as well as assessment of unwanted side effects [31].

Activation of the ROCK cascade has not been previously assessed in diabetic patients. Here, in the T2D patients receiving glucose-lowering drugs, both increased phosphorylation of the upstream molecule JAK-2 and of the downstream MAPK (p38 MAPK) were observed for the first time in this clinical context.

p38 MAPK plays important roles in regulating glucose metabolism in skeletal muscle and in adipose tissue [32]. Studies with cultured skeletal muscle cells indicate that p38 MAPK enhances insulin and exercise or AMPK-induced glucose uptake by activating GLUT4 [33, 34]. Besides, MAP kinases, such as the downstream ROCK cascade molecule p38-MAPK, have a role in the development of cardiac hypertrophy, remodeling, and contractile dysfunction (35 30). Increased cardiac p38-MAPK expression is associated with reduced contractility and the development of cardiomyopathy in mice [35]. In rats with cardiac remodeling induced by endurance exercise, the ROCK inhibitor fasudil reduces cardiac hypertrophy, remodeling and cardiac p38-MAPK levels [36]. Interestingly, PMBCs p38-MAPK phosphorylation levels and cardiac and aortic wall p38-MAPK phosphorylation levels secondary to ROCK activation have been observed in normotensive and in hypertensive rats [23, 26]. Even though LV systolic function assessed by LV ejection fraction was normal in the current T2D patients, this is the first observation of increased p38-MAPK phosphorylation in PMBCs in T2D patients, associated to ROCK activation, specifically in patients with high blood glucose levels, concentric remodeling and evidence of diastolic dysfunction (lower E/A ratio and increased A wave velocity).

In preclinical studies, the upstream JAK 2 protein has a known role in the development of diabetic nephropathy. High glucose levels augment ANG II-induced activation of JAK2 in renal glomerular mesangial cells and in vascular smooth vascular cells [37–39]. Interestingly, simvastatin modulates the detrimental effects of high glucose/ANG II in diabetic kidney glomeruli both in vitro and in vivo by preventing the high glucose and ANG II-induced geranylgeranyl-dependent activation of Rac or/and Rho and thereby blocking the activation of JAK2 and both STAT1 and STAT3 [40]. Besides, inflammation signaled by Janus kinases (JAKs) promotes progression of diabetic kidney disease [41]. In diabetic mice, enhanced expression of JAK2 selectively in glomerular podocytes increases pathological characteristics of diabetic kidney disease [42]. Recently, in a randomized clinical study in T2D patients at high risk for progressive kidney disease, baricitinib, an oral, reversible selective inhibitor of JAK1 and JAK2 reduced albuminuria [41]. In keeping with the abovementioned observations, in the current patients under treatment and with increased microalbuminuria, JAK activation (phosphorylation) compared to control subjects was observed.

Plasma MDA levels were measured here as an oxidative stress biomarker, more precisely a lipid peroxidation marker, and were higher in T2D patients compared to controls. Oxidative stress in diabetes exacerbates atrial structural and electrical remodeling, promotes Ca^2+^ mediated triggers and the initiation of atrial fibrillation [43]. MDA levels are elevated in patients with metabolic syndrome [44, 45]. Possibly, higher MDA levels in the T2D patients could originate from lower plasma antioxidant enzymes levels (not measured here) and to the over active leukocytes [44] secondary to ROCK activation.

Regarding T2D, Rho kinase activation and inflammation, T2D is a chronic low-grade inflammation state [46]. Several circulating inflammatory biomarkers are elevated in T2D patients under treatment compared to controls and are associated to a hypercoagulable state and vascular dysfunction [47]. Increased IL-6 (as well as IL-18) circulating levels in T2D patients under treatment could trigger an increased extracellular matrix proteolysis, which may modify vascular structure and function [48]. Interestingly, in the T2D patients under treatment (but still with hyperglycemia) we observed increased levels of ICAM-1, VCAM-1 and IL-8 in PBMCs associated to ROCK activation whereas their circulating of both IL-6 and IL-8 were similar to controls. The ROCK pathway plays a role in adhesion molecule expression and inflammatory cell infiltration in glomerular endothelial cells induced by advanced glycation end products [49]. Besides, ROCK activation mediates regulation of inflammatory signaling pathways in human retinal cells exposed to high-glucose as well as in diabetic mice [50].

### Limitations

In this cross sectional study we used two groups, controls and T2D receiving glucose-lowering drugs (or insulin), antihypertensive drugs and some of them statins as well. It would be most interesting to assess ROCK levels in T2D patients without treatment. Nevertheless, in the only available study addressing ROCK activation in PBMCs in T2D patients 75.6% of them were receiving metformin, 58.5% gliclazide and 26.8% a DPP-4 inhibitor [12]. Besides, 63.7% were receiving ACE inhibitors or ARBs, 63.6% calcium-channel blockers and 68.4% statins [12]. Even though all these drugs may reduce ROCK activity, our results are consistent with those findings (compared with the control subjects, ROCK activity was significantly increased in T2D patients). Besides, since the T2D group was treated with glucose-lowering drugs (most of them with metformin) and some of them with insulin and they still had hyperglycemia, it would have been relevant to compare our current findings with a group of T2D patients with well controlled blood glucose levels.

## Conclusions

T2D patients under current treatment with antidiabetic and antihypertensive drugs as well as with statins have significantly increased ROCK activation in their PMBCs, also with increased phosphorylation of upstream and downstream cascade proteins. ROCK activation levels were correlated here with current blood glucose levels. In these patients, increased levels of the proinflammatory molecules VCAM, ICAM-1 and IL-8 were also found in their PMBCs despite pharmacologic treatment along with increased plasma angiotensin II and MDA levels. It is possible to hypothesize that specific ROCK inhibition might have an additional role in the prevention and treatment of T2D.

## Data Availability

The datasets used and/or analysed during the current study are available from the corresponding author on reasonable request.

## Abbreviations

EDTA: Ethylenediaminetetraacetic acid
ERM: Ezrin, radixin, moesin
GLP1: Glucagon-like peptide 1
HbA1c: Glycated hemoglobin A1c
ICAM-1: Intracellular adhesion molecule 1
IL-6: Interleukin 6
IL-8: Interleukin 8
JAK: Janus kinase
JNK: c-Jun N-terminal kinase
LV: Left ventricle
MAPK: Mitogen-activated protein kinase
MLC-2: Myosin light chain 2
MYPT-1: Myosin phosphatase target subunit 1
NFκB: Nuclear factor κB
PBMCs: Peripheral blood mononuclear cells
ROCK: Rho kinase
T2D: Type 2 diabetes mellitus
VCAM-1: Vascular cell adhesion molecule 1
